# Costs and Willingness to Pay for Pit Latrine Emptying Services in Kigali, Rwanda

**DOI:** 10.3390/ijerph16234738

**Published:** 2019-11-27

**Authors:** Zachary Burt, Rachel Sklar, Ashley Murray

**Affiliations:** 1 Athena Infonomics, Rockville, MD 20850, USA; 2 Department of Environmental Health Sciences, University of California, Berkeley, CA 94720, USA; 3 Independent Consultant, Northampton, MA 01060, USA; murray.ash@gmail.com

**Keywords:** fecal sludge management, FSM, on-site sanitation, pit latrines, informal settlements, sanitation, formalization, willingness to pay, cost of service

## Abstract

Kigali, Rwanda lacks a centralized sewer system, which leaves residents to choose between on-site options; the majority of residents in informal settlements use pit latrines as their primary form of sanitation. When their pits fill, the pits are either sealed, or emptied; emptying is often done by hand and then dumped in the environment, putting the residents and the broader population at risk of infectious disease outbreaks. In this paper, we used revealed and stated preference models to: (1) estimate the demand curve for improved emptying services; and, (2) evaluate household preferences and the willingness to pay (WTP) for different attributes of improved emptying services. We also quantify the costs of improved service delivery at different scales of production. The study included 1167 households from Kigali, Rwanda across 30 geographic clusters. Our results show that, at a price of US$79 per pit, 15% of all the pits would be emptied by improved emptying services, roughly the current rate of manual emptying. Grouping empties by neighborhood and ensuring that each truck services an average of four households per day could reduce the production costs to US$44 per empty, ensuring full cost coverage at that price. At a lower price of US$24, we estimate that the sealing of pits might be fully eliminated, with full coverage of improved emptying services for all pits; this would require a relatively small subsidy of US$20 per empty. Our results show that households had strong preferences for fecal sludge (FS) treatment, formalized services (which include worker protections), and distant disposal. The results from the study indicate a few key policies and operational strategies that can be used for maximizing the inclusion of low-income households in safely managed sanitation services, while also incorporating household preferences and participation.

## 1. Introduction

Like many cities in Sub-Saharan Africa, Kigali, Rwanda is experiencing an urbanization rate that is over twice the global average [1]. This has led to the emergence of large and dense informal settlements. Urban planning for sanitation, and for fecal sludge management (FSM), in particular, has struggled to keep pace with population growth. Kigali has no sewer system. Instead, households rely on onsite sanitation. Septic tanks are the norm in wealthier houses, while poorer houses often construct dry pit latrines. Currently, the vast majority (91%) are pit latrines [2]. Additionally, with Kigali’s growing urban population, there is limited and dwindling space on household plots for sealing and then digging a fresh pit. In 2013, Tsinda’s survey of households in Kigali found that only 2% had ever been de-sludged, and, of those, 34% reported taking the resulting fecal sludge to the municipal landfill, while the remainder reported disposing sludge ‘indiscriminately in dumpsites’ [3]. In another study, in Kigali, Kisumu and Kampala both mechanical and manual emptying services were observed to be available, although both such services were relatively limited in Kigali. In the same study it was observed that manual emptiers did not transport the waste, but disposed of it by either dumping it directly into the nearby storm drain, or burying it on site, and that there were no available treatment services for mechanical collectors in Kampala and Kigali [4]. In a third study, in Kampala, 59% of slum households with pit latrines stated that they emptied it when full, while 11% said that they would seal it and dig a new pit, and 30% either said that they did not know what they would do, or left it up to their landlord [5].

Without the development of alternatives, as new pits fill, and sealing becomes more difficult, the volume of fecal sludge that is ‘indiscriminately’ disposed will grow in proportion to the population. Many low-income households in dense areas are logistically difficult to access with regular exhauster trucks (vacuum trucks) due to steep slopes and unpaved, narrow roads. This combined with the high prices for exhauster truck services relative to the low incomes of households has resulted in limited formal emptying services being delivered in informal neighborhoods.

Efforts aimed at encouraging safer collection and treatment of Fecal Sludge (FS) run the risk of increasing the cost of service provision, potentially moving prices beyond what some households are willing to pay, or can afford. Interventions that are aimed at improving FS services risk failure if estimates of willingness to pay and the cost of service are not incorporated into their design from the beginning. The costs of FS collection have been reported in a handful of recent studies, although it is not well documented in the research literature in general (see Table 1) [6,7,8,9]. Fuel costs are the largest expense for service providers, and the prices are dependent on the volume of the requested truck, the distance between the exhauster truck and the pit, and the amount of solid waste that are found in the pit. Higher prices were also charged for dirty latrines, latrines distant from a drainage channel, and for empties that were performed during the dry season [7].

Estimates of the willingness to pay (WTP) for FS collection are also not well documented in the research literature (see Table 2) [9,10,11,12]. Balasubramanya et al. estimated the willingness to pay for FS collection, roughly half the cost of the cheapest possible service delivery option, according to their cost models [10]. Harder et al. estimated the WTP for FS collection services, being charged as a monthly fee, for regular, formalized, municipal septage services [12]. In urban Senegal, Scott et al. found that tenants were less likely than owner-occupied households to invest in sanitation, but just as likely to pay for emptying [13].

Lowering prices and increasing affordability can be achieved to a certain extent through private initiatives that create either greater cost efficiency of services or alternative revenue generation through resource recovery. Resource recovery has been described as a type of internal subsidy that might be used to lower the prices and increase access [14,15,16]. One potential strategy for achieving greater cost efficiency involves the use of transfer stations; Kennedy-Walker et al. found that network analysis was potentially useful in minimizing the costs through the strategic placement of transfer stations in the system of collection and transportation of fecal sludge [17]. This paper does not explore resource recovery or strategic placement of transfer stations; instead, we explore another alternative: demand aggregation. We define demand aggregation as the clustering of multiple empties among neighbors. Demand aggregation is one possible way to spread the fuel costs from a single trip over two or more houses, while also increasing the volumes that are transported on each trip. Ensuring that trucks are working in a specific geographical area on a given day, rather than serving geographically dispersed households, has the potential to change the economics of pit emptying by reducing the fuel costs and the time spent per empty. Mandatory regular desludging for all onsite units at predefined regular intervals is a directly managed, top-down way to potentially create clustered empties. However, it is an open question as to whether efforts to organize FS collection in neighborhood clusters are effectively done through fiat, while using government services. There are data and a growing international consensus that shows that private markets are often a viable service delivery mechanism, especially where government services are limited [18]. Ultimately, demand aggregation should be evaluated in comparison to other cost minimization strategies and to the potential of alternative revenue generation from resource recovery; we present this paper as a small contribution to that larger goal.

This study is a gap analysis between the WTP and the cost of service delivery for improved, formalized FS collection, operating in Kigali, Rwanda. Pit Vidura is a social enterprise that delivers formalized, improved pit latrine emptying and FS transport services to low-income households in informal settlements in Kigali. The improvements, as compared to other pit emptying services, include branding, uniformed workers using protective gear, effective treatment of FS, distant disposal, and the reuse of FS. In this paper, we construct a cost model of Pit Vidura’s formal pit emptying services, characterize the local market for sealing and emptying in informal settlements of Kigali, and then compare them both to estimates of willingness to pay (WTP) for Pit Vidura’s services. We used discrete choice, stated preference models to determine the household drivers of demand, and to evaluate both the tenant and landlord preferences, as well as the WTP for different attributes of the emptying service. We focused on identifying WTP for the key aspects that distinguish Pit Vidura. In addition, we incorporated the possibility of group empties into our cost analysis and our WTP protocol on the hypothesis that serving geographically clustered houses could provide cost savings, provided that households were willing to voluntarily coordinate the timing of their empty.

## 2. Materials and Methods

### 2.1. Household Sampling Protocol

This study was conducted in ten of Kigali’s 35 sectors. We selected these sectors based on the presence of high numbers of people living in dense informal settlements, and, therefore, unlikely to use formal sanitation services. Pit Vidura had previously conducted marketing and pit emptying services in two sectors; we avoided those sectors and the neighboring sectors. Sectors with a high proportion of high-income households, which are generally located on regular roadside plots accessible by exhauster trucks, were also deemed to be ineligible for this study, because they were unlikely to use the services provided by Pit Vidura. We chose 30 villages distributed across the ten selected sectors to be a part of the study sample. Villages that were adjacent to each other were not selected and all villages that were selected had an Executive Secretary who endorsed the study. All of the households in selected villages were eligible to participate in the study if they (1) had a latrine and (2) had at least one household member over the age of 18. Enumerators were sent to a central point in each village as a pair. From the central point, each enumerator in the pair went in opposite directions, approaching every third house along the way, going to the nearest neighbor in the case of refusal, until they each had 20 households. In many instances, this sampling method caused them to reach the ends of the village before fulfilling this quota, in which case they doubled back and returned to the point of origin through other street paths. In total, we distributed coupons and conducted baseline surveys to 1167 households, as some villages had less than 40 households for receiving coupons.

### 2.2. Revealed Preference Study Design

Each village was randomly assigned one type of coupon, and all of the participants in that village received the same coupon. Each coupon included a volumetric price for an individual empty, as well as a volumetric discount for a group empty; see Table 3 for prices, see supplementary Figure S1 for an example coupon (exchange rate: US$1 = RwF 900). These baseline prices ranged from 38% of the retail value to 150% of the retail value. As an example, the price of a typical empty (2000 liters) was also printed on the coupon. The coupons included a validity date (90 days from the start of the study), a serial number to avoid duplication, and an explanation of how to use them. Each participating household was given one coupon and then assigned a household identification number. Serial numbers were checked against identification numbers when Pit Vidura services were requested. The prices varied across coupon types, but everything else was constant across all coupons.

### 2.3. Stated Preference Study Design

We conducted a stated preference survey of WTP for improved pit emptying services, while using a discrete choice framework, during our follow-up surveys of households and landlords. We included eight attributes of FS collection services in the discrete choice survey: volumetric price, pit fullness, formalized services, speed of response, distance of disposal and treatment, flat fee, and sealing. Six prices were included in our survey; only two levels were included for all other attributes (see Table 4 for all levels and attributes). Enumerators were given a set of cards containing all possible combinations of the non-price attributes—32 cards in total, numbered one through 32. Two additional cards (numbered 33 and 34) were included in the set, one with the option of sealing and the other with the option of an informal empty, both of which only cost a flat fee. Each choice set included two random cards from the set of one through 32, plus cards 33 and 34, with random prices being chosen for both volumetric and flat fee options. Each participant was shown three choices sets, and then asked to indicate which two options in each choice set were their first and second preferences. The choice-sets were randomly assigned to participants via a household identification number before going to the field. All obvious-preference choice sets, where higher prices were matched with worse attribute-levels, were eliminated from the pool before assignment.

### 2.4. Data Collection

Cost data were taken directly from expense reports that were associated with each household served. Two kinds of cost data were collected: operational expenditures (opex) and overhead expenditures. Opex only included the expenses incurred by each additional pit emptying, while overhead included all other costs, which were a bulk amount divided over all empties. Opex costs are reported for all households that were served during the trial period. Overhead costs, other than marketing, were proportionally attributed based on the number of households served in the trial (versus non-trial households served during the three-month trial period). All of the cost data were taken from Pit Vidura expense reports.

This study presents two costing models, one derived from a rented flatbed truck and the other from a purchased exhauster truck. During the trial, Pit Vidura provided household emptying services while using a rented flatbed truck. The daily rental was a flat fee that included fuel, driver, and the truck itself. Distance traveled is not considered in our analysis, a cost that would reach greater efficiencies by group empties with the exhauster truck, since the same flat rate applied regardless of distance or fuel use. The flatbed truck that was used during the trial period had a holding capacity of 100 50 L barrels, or 5 m^3^ in total, which is sufficient capacity for handling at least two empties. The exhauster truck had a capacity of up to 10 m^3^, which is sufficient for handling up to four empties.

Labor costs in both the flatbed and exhauster truck scenarios included the evacuation of FS, transportation, discharge at the treatment facility, and cleaning all equipment and barrels following service completion. The salary of the driver was included in the labor costs in the exhauster truck scenario. The tipping fee was a per-trip fee paid to the city of Kigali for discharge of FS at the sanctioned disposal site. During non-trial normal operations, a commission is paid to salespeople for acquiring customers in the field. These salespeople were not used for households that were part of the coupon trial. However, the same average cost for sales commissions was applied to the trial period, since enumerator visits were considered to be substitutes for this type of direct marketing; they explained the service, left households with coupons, and answered any immediate questions, as a sales person would. See Table 5 for the categorization of costs in the flatbed and exhauster truck scenarios.

At the start of the study, households were given their coupons along with a short survey (Survey 1) to collect basic contact information and information regarding household latrine practices (e.g., type of emptying behavior, frequency service use, and the responsible payer for this service). All of the participating households received one text per week for the duration of the 12-week trial that reminded households to take advantage of their special offer. Households that requested services were also given a follow-up survey (Survey 2) to gain more information on household characteristics and socio-economic status (SES), features of toilet serviced, and household satisfaction with existing FSM services. If landlords were deemed to be fully responsible for pit maintenance in Survey 1, then the landlord of the household was also given a survey (Survey 3) that was aimed at gaining much of the same information as Survey 2. Following the 90-day study period, a random sample of 100 households that chose not to use their coupons were administered either Survey 2 or 3. Survey 3 was completed over the phone for landlords that did not live nearby. All data were recorded while using the Open Data Kit platform on Android phone devices.

### 2.5. Data Analysis

We calculated the average costs under different cost scenarios and for different scales of service delivery. Summary statistics were calculated for our market assessment analysis, including both emptying and sealing services, and the average retail prices that are being charged for services. Stated preference data are analyzed while using a discrete choice model, directly giving WTP estimates for specified attributes. Revealed preference data are analyzed using a logistic regression model, in order to build a model for demand forecasting. Plugging our market assessment results into the forecasting model then created WTP estimates.

In addition to summary statistics on survey data, we also estimated the size of the pit maintenance market while using two different methods. The first method used reported data on the fullness of pits; calculated the percentage of households that reported having a full pit to estimate the probability of having a full pit (P_1_). The second used reported data regarding the frequency of filling, by taking the inverse of filling frequency:

(1) $$Prob\left(Pit=Full\;|\;T\right)=T*\;R_{i}{}^{-1}$$

(2) $$P_{2}=\;\frac{1}{N}\overset{N}{\underset{i}{\sum }}T*\;R_{i}{}^{-1}$$

where *P*_2_ is the proportion of pits that are likely to be full, *R_i_* is the filling frequency in months, and T is the number of months. This assumes that our filling frequency data are representative of pits in general and do not take the age of the pits into account.

Cluster randomization of coupon prices allowed for us to create a statistical model of uptake, in order to model WTP while using a revealed preferences approach. We modeled two dependent variables while using logistic regression: (1) any request for emptying services; and, (2) any request for group emptying services. We only included service requests for Pit Vidura services, coming from our original enrollment of 1167 households, plus the 12 households that did not receive a coupon, but were part of a group empty along with a coupon-holding neighbor (making the total N = 1179). Any sealing or emptying services that were provided by other parties to these households are not included in these models. For Yi (I = 1, 2, …, n), as one of these two dependent variables, follows a Bernoulli probability function that takes on the value 1 with probability πi and 0 with probability 1–πi, and then for i = 1, 2, …, n.

(3) $$\pi_{i}=\;\frac{1}{1+\;e^{-\left(\alpha +\;\beta_{k}x_{k,i}\right)}}$$

where α is a constant, $x_{k,i}$ are k independent variables for individual households i….n, and βk are coefficients estimated for k independent variables. Rearranging the equation, such that the dependent variable estimated is the log-odds ratio:

(4) $$ln\left(\frac{\pi_{i}}{1-\pi_{i}}\right)=\;\alpha +\;\beta_{k}x_{k,i}$$

which is then estimated while using maximum likelihood techniques. In our model we included variables ($x_{k,i}$) for the volumetric price charged for an empty, the volumetric discount offered, whether the household was solely or partially responsible for pit maintenance (vs. if the landlord was solely responsible) and a categorical variable of reported pit fullness (either nearly full, or full, vs. less than nearly full).

Stated-preference discrete choice models have been used to model consumer preferences across multiple disciplines, such as transportation [19] and environmental services [20,21]. We employ a multinomial logit specification to model the choices of the subjects and infer how they value different attributes relative to each other. For a multinomial logit model with a linear-in-parameters model specification, the utility of alternative j over choice-set t as perceived by individual n, denoted untj, is given, as follows:

(5) $$u_{\mathrm{ntj}}={\beta x}_{\mathrm{ntj}}+\varepsilon_{\mathrm{ntj}}$$

This equation was re-arranged after initial modeling efforts, such that WTP could be directly estimated:

(6) $$u_{\mathrm{ntj}}=\beta_{p}*(p_{\mathrm{ntj}}+{\beta^{\prime }x^{\prime }}_{\mathrm{ntj}})+\varepsilon_{\mathrm{ntj}}$$

where $x_{\mathrm{ntj}}$ is a vector of explanatory variables, such as the attributes of the options presented and the characteristics of the individual; $p_{\mathrm{ntj}}$ is a vector of prices; ${x^{\prime }}_{\mathrm{ntj}}$ is the vector of explanatory variables, minus price; β is a vector of taste parameters; $\beta_{p}$ is an estimate of the marginal impact of price; $\beta^{\prime }$ is a vector of WTP estimates for each of the taste parameters; and, $\varepsilon_{\mathrm{ntj}}$ is the stochastic component of the utility. $\varepsilon_{\mathrm{ntj}}$ denotes that which is unobserved by the analyst or purely random, assumed to have an identical and independent Gumbel distribution across all ε, with location zero and scale one across alternatives, choice-sets, and individuals. Let $y_{\mathrm{ntj}}$ denote the choice indicator, being equal to one if individual n chooses alternative j over choice-set t, and zero otherwise. Under these assumptions, and further assuming that individuals are utility maximizing, the probability that individual n chooses a sequence of choices $y_{n}=❬y_{n11},\;\ldots ,y_{\mathrm{nTJ}}❭$, where T denotes the number of choice-sets that are faced by a single individual (equal to three in our case), J denotes the set of alternatives in the first ranking for any given choice-set (equal to four in our case), and J’ denotes the set of alternatives in the second ranking for any given choice set (excludes the alternative chosen as the first ranking) may be given, as follows:

(7) $${\mathrm{Pr}}_{y}\left(y_{n}|x_{\mathrm{ntj}}\right)=\overset{T}{\underset{t=1}{\prod }}\overset{J}{\underset{j=1}{\prod }}{\left[\frac{\exp\left({\beta x}_{\mathrm{ntj}}\right)}{\sum_{j=1}^{J}\exp\left({\beta x}_{\mathrm{ntj}}\right)}\right]}^{y_{\mathrm{ntj}}}{\left[\frac{\exp\left({\beta x}_{{\mathrm{ntj}}^{\prime }}\right)}{\sum_{j^{\prime }=1}^{J^{\prime }}\exp\left({\beta x}_{{\mathrm{ntj}}^{\prime }}\right)}\right]}^{{y_{\mathrm{ntj}}}^{\prime }}$$

The unknown model parameters (β) are the taste parameters (the attributes included in our choice sets), and these are estimated via maximum likelihood estimation while using the free discrete choice estimation software Biogeme [22]. The reader is referred to Train (2009) for more information on the specification and estimation of multinomial models [23].

## 3. Results

### 3.1. Market Analysis

**Figure 1:**
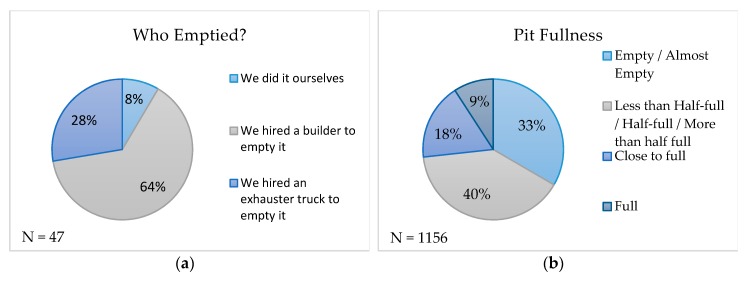

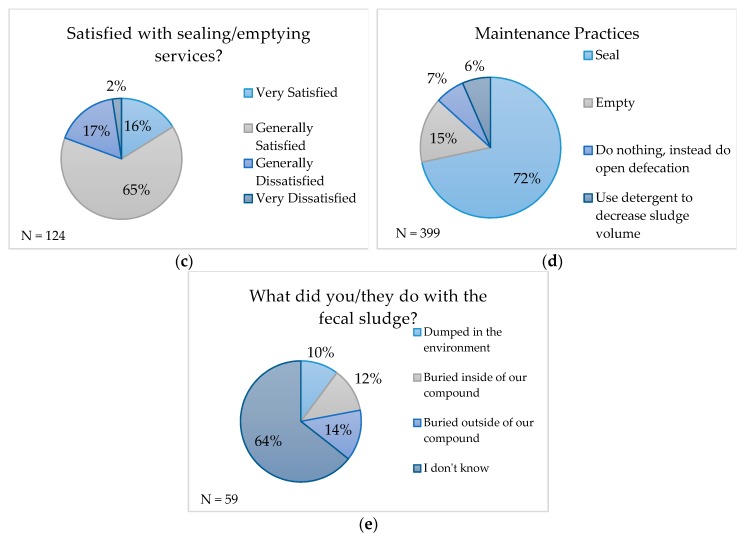
Market analysis regarding pit fullness, pit maintenance practices, and customer satisfaction: (**a**) the service provider who emptied the pit, among households that had emptied their pit in the past; (**b**) the relative fullness of pits, as reported by households; (**c**) customer satisfaction, among households that have emptied or sealed in the past; (**d**) pit maintenance practices, among households that had a full pit in the past; and, (**e**) FS disposal practices, among households that had emptied in the past.

We only included a short list of questions in Survey 1, in order to maximize our sample size; these questions had a maximum N of 1167. We included many more detailed questions regarding pit maintenance practices and the cost of services in the follow-up surveys (Survey 2 and 3); these had a maximum N of 157. In our sample, approximately 1/3 of respondents lived in a house with only one household, while 2/3 lived in a shared house, with multiple households living in one place. We explicitly defined household for the respondents: ‘…the people living with you in the same place, sharing meals and living expenses. This is probably people you are closely related to’. Using this definition, our sample had an average of five rooms and six people per household. People have been living and using a latrine for many years, but only a small number had emptied their pit before, despite 43% having had a full pit in the past (see Table 6). This is likely because sealing is, by far, the preferred pit maintenance method (see Figure 1). Sealing includes both sealing the old pit and digging a new one; it can use the same super-structure or can include building a new one, if the new pit is not nearby. Few people remembered the cost of sealing, but it averaged US$ 109 (± 51.3) across all types of sealing. The mean reported cost of emptying was US$ 52 (± 14), which includes both formal and informal services (see Table 6). The fact that sealing is preferred, despite the higher price, implies that it has some desirable attributes when compared to emptying; a better understanding of what those attributes might be is worth greater scrutiny. Historically, sealing has remained the most common practice in informal settlements—it is the only alternative to manually emptying pits. Most formal service providers do not extend their services to informal areas due to difficulties in access as well as payment collection. However, it is important to note that sealing a pit and building a new pit is no longer practical in crowded urban areas, and under forthcoming government regulations, will be prohibited. The need for affordable alternatives to manual emptying is dire.

A minority of households reported having full (9.2%) or almost full (17.6%) pits (see Figure 1). Only 9.4% of households that had full or almost full pits requested Pit Vidura services. However, this aggregated demand does not account for the effect of our price randomization; it is necessary to model the relationship between price and demand to characterize user preferences and identify factors that influence demand, such as pit fullness (see our revealed preference results and analysis). P_1_ in our survey was 9.2%, while P_2_ was estimated to be 10.7%, for an average of 9.95%. 15% of pits were reported to be emptied when full (see Figure 1), yielding an estimate that 1.5% of all pits are emptied every three months. For those households that emptied, only 28% reported that they hired an exhauster truck service in the past (see Figure 1). Therefore, we estimate that before Pit Vidura enters an area, 0.42% of all pits are emptied while using formalized emptying services. 

Informal emptiers universally work without protective equipment and lack formal training in waste management; they typically use buckets, shovels, and picks. After an empty, 10% reported dumping in the environment or a drainage canal, but a full 64% who answered this question reported ‘I don’t know/I don’t want to say’ (see Figure 1). Dumping is illegal and it carries a stiff fine, so it is likely that the true percentage of people dumping after an empty is much higher; but even this level of reporting indicates that it is likely a typical option that is practiced by many households.

### 3.2. Cost Analysis

Unit costs are shown per empty, for the flatbed truck in Table 7, and for the exhauster truck in Table 8. During the trial, the flatbed truck was operating at under-capacity and served only one customer on most days. We present projections showing the costs over a range of minimum and maximum empties for both of the trucks. We estimate the cost per empty ranges between US$61 and US$191, depending on the number of households served per day and the capacity of the truck used. If the 5 m^3^ rental truck is used at max capacity, the cost of delivering services is US$96/empty, while the cost of delivering services in the 10 m^3^ owned truck drops to US$61/empty. In the flatbed truck scenario, the most expensive opex cost was the truck rental, while it was fuel in the exhauster truck scenario. In both scenarios, the most expensive overhead cost was management staff.

For days in which two or more empties are completed per truck, the cost per empty using the exhauster truck is lower. At four empties per day (the maximum capacity of the exhauster truck), the cost of service delivery while using the exhauster truck is US$61/empty, roughly 2/3 the cost of services using the flatbed truck at two empties per day (the maximum capacity of the flatbed truck). This points to an important driver of cost efficiency: fitting as many empties in a single load, coming from the same location, and before driving to the dump site. While the flatbed rental might be enough to handle a group empty consisting of two pits, a larger capacity exhauster truck would be needed to empty three or more pits.

A significant difference in the labor requirements of the exhauster truck and the flatbed truck exists. This is because, while using the exhauster truck, only two laborers are needed to complete 1–2 empties versus a requirement of five laborers for flatbed operations. In the case of the flatbed, extra labor is required to transport barrels from the pit to the truck, while, in the exhauster truck case, an extended hose is used to move sludge from the pit to the roadside. In the latter case, labor at the pit is used to man pumps, remove trash from the pit, and clean the work site. In the case of the exhauster truck, workers are incentivized with an additional bonus of between 40–60% if three or four empties per truck are completed. Geographic clusters of households that are ready to serve by a single truck on a given day are necessary in order to fill each truck to maximum capacity and avoid the time and costs associated with driving to geographically dispersed households. As such, the response to the discount offered for clustered empties as part of the revealed preferences study design is of particular interest.

### 3.3. Willingness to Pay—Revealed Preference Analysis

As shown in the Market Analysis section, the demand for pit emptying services in a given three-month window is a small proportion of households overall. Therefore, demand characterization can be disproportionately impacted by the gaps in the data for households that requested services. Unfortunately, our data collection during follow up was incomplete, due to logistical constraints. Coupon holders requesting group empties were not required to groups themselves with other coupon holders. Of the 62 households that requested Pit Vidura services, we do not have the coupon number for three of these ‘neighbor’ households; these were entirely dropped from our analysis. For an additional nine ‘neighbor’ households, we do have the coupon numbers which were used for their empty, but since they requested services as part of a group empty with a coupon holder, they were not given Survey 1, and we do not have pit fullness data (predating their empty). There was also one house that requested services, received Survey 1, but did not know their pit fullness. These 10 households are excluded from models that include the pit fullness variable, but included otherwise. Lastly, of the 59 households that requested services and for which we have coupon numbers, only 45 received emptying services during the study period; for the 14 households that requested but did not receive service, we are not able to discern whether they requested group empties or individual empties. For these households, we have assumed that they requested individual empties. This assumption should not affect our total uptake models, but it might have depressed our estimates for the impact of the group discount prices.

In our logistic regression model, we converted the volumetric price in Rwandan Francs to a price per average empty, in US dollars. The average volumes of empties during this study were 1200 Liters; the price per empty was calculated based on this volume. We estimated the logistic regression model for service requests against the individual empty price and the group discount (see Model 1 and Model 2 in Table 9). We then regressed all of the relevant household characteristics that were collected during Survey 1 against the log-odds of requesting services, in combination with the individual empty price and the group discount. Those characteristics included (1) whether the household was responsible for pit maintenance costs; (2) pit fullness (both ‘almost full’ and ‘full’ categories were included); (3) the pit filling frequency; (4) whether they have had a pit emptied before; (5) whether they have had a pit sealed before; and, (6) whether they have had a pit completely fill before. The first two characteristics yielded statistically significant coefficient estimates; they are further explored below (see Table 9). The models that were estimated for the remaining four household characteristics can be found in the supplemental information (see Supplementary Table S1).

**Figure 2:**
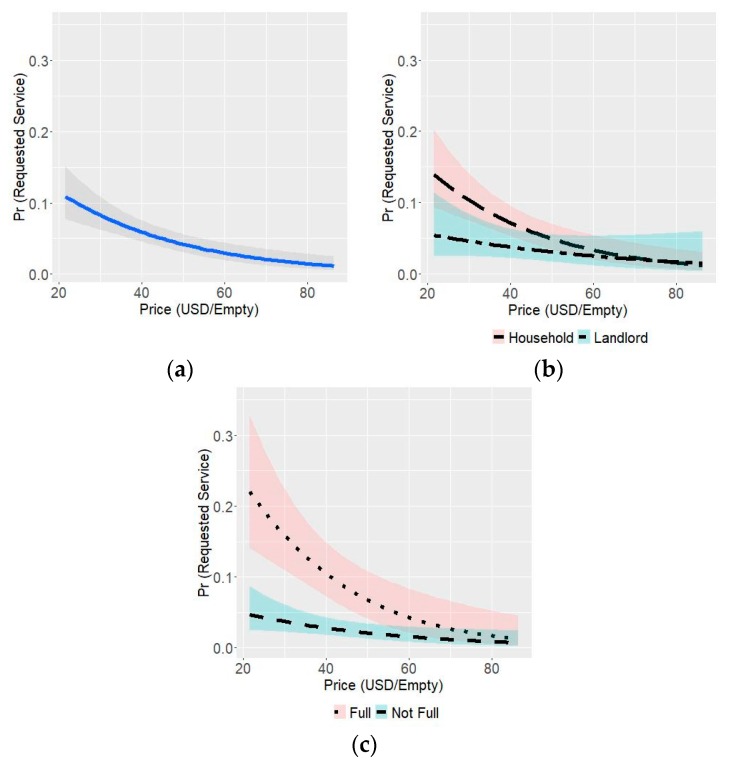
Graphical comparison of Model 1 (**a**), Model 3 (**b**) and Model 4 (**c**), with 95% confidence intervals.

The estimated coefficients for the individual price per empty were robust across all models, lending significant confidence to our estimate. The estimated coefficient for group discounts was statistically significant and consistent across models, both with and without the household responsible dummy variable. When pit fullness was included, the estimate shifted and it was no longer statistically significant. The correlation coefficient between these variables, and the correlation coefficient with residual errors was low (<0.07 in all cases). The same pattern was observed for the household responsible variable. The estimated coefficients for pit fullness variables were robust and statistically significant across all of the estimated models. We ran the same independent variables against group empty requests (see Table 9 and Tables S2 and S3 in the Supplementary for additional model variations). We saw a very similar pattern, similar estimates for the impact of price, and slightly stronger relationship with the group discount, household responsibility, and full pits. Graphical comparisons of Model 1, Model 3, and Model 4, with 95% confidence intervals, can be found in Figure 2.

We plugged in a few key price levels into Model 1 to forecast demand, along with 95% confidence intervals. Table 10 shows the demand forecasts and correspond to our estimate of the current demand for formal, mechanized emptying services (0.4% of households), all emptying services (1.5%), half of all full pits (5%), and all full pits (10%), for a given three-month window of time. At a price of US$79, the demand for Pit Vidura would reach the total for all current emptying services. This is US$27 more than the average cost of emptying found in Survey 2 and 3 (see Table 6). The demand forecasts for group empties had large uncertainty, but they generally showed that, at discounts of US$7.2 and US$14.4 per empty, approximately 1/3 of total demand and 2/3 of total demand, respectively, would be for group empties (see Supplementary Table S4).

### 3.4. Willingness to Pay-Stated Preference Analysis

Of the total 1167 households that received coupons, 62 requested Pit Vidura emptying services. An attempt was made to administer Survey 2 with these households, and with their landlords Survey 3, as appropriate. Of these, 50 were successfully surveyed, plus a random selection of 107 households that did not request services, for a total sample size of 157 households that were included in the stated preference survey.

Table 11 presents the coefficient estimate for the marginal impact of price, and the modeled estimates of WTP for all taste parameters from the stated preference analysis, in US$. All of the attributes were statistically significant, except for ‘faster response’ and ‘empty only when full’—indicating that households were, on average, indifferent to the faster response time that we presented to them, and amenable to the idea of group empties (that may require coordinated emptying, before some pits are full). The coefficient for flat fees was negative, indicating a preference for volumetric pricing. The WTP for treatment was almost double the WTP for either ‘branded + work protections’ or ‘distant disposal’, but it was slightly less than the WTP for sealing (instead of emptying). All of the WTP estimates should be considered as a premium that households would pay on top of a base price—in this case the base price would be informal emptying (that has none of the attributes included in the model). As stated above, our market assessment estimate of the average price of emptying services in informal or low-income areas of Kigali was US$52 at the time of the study.

## 4. Discussion

In our assessment of the FSM system in Kigali’s informal and low-income areas, we found a nascent fecal sludge management system, operated by private actors in both formal and informal sectors. When the pits are full, most of the households reported that they sealed their pits; indeed, this preference was also born out in our stated preference model results. This option may pose a lower risk to environmental health than emptying, when fecal sludge treatment is not ensured before disposal. However, sealing is by no means an urban panacea: it might endanger water supplies, whether they are in nearby, leaky pipes, in shallow aquifers, or in downstream surface water sources. Furthermore, sealing requires another pit to be dug; the number of times this is possible, without emptying the contents of older pits, is limited by the space available. This indicates that, while emptying may currently play a minor role in FSM, its influence on public health and environmental pollution will only grow over time.

While most governments provide heavy subsidies for storm water drainage, sewerage, and even wastewater treatment where it exists, the regulation, oversight, investment, and subsidy of FS management have been limited. Services are almost always provided by the private sector, and often by informal operators. Private actors have filled this gap, often by taking on work that others would find dirty and disgusting, at low profit margins and little thanks; yet, market competition can only reduce prices up to a point, and that point is too often out of reach for low-income households. This leads to services that cut corners on worker protections and environmental health, in many cases. Finding ways to meet the cost of emptying pits and septic tanks when servicing low-income clients remains a central challenge.

Informal emptying services in Kigali are often manually done, with no protective gear. The disposal of FS often occurs near the home, and with no treatment of the FS before disposal. Pit Vidura addresses these gaps, but it is an open question whether Pit Vidura can survive on its own as a private enterprise. We analyzed the costs of service provision and the WTP for improved FS collection, in order to create scenarios for the supply and the demand for improved fecal sludge collection services in Kigali. The goal of creating different scenarios is to identify the key levers and potential points of intervention that maximize the numbers of households both choosing to empty their pits and engaging improved emptying services. This goal is in line with SDG 6 as well as broader public health aims.

We found that scale was a key determinant of costs—providing four empties per day with the larger capacity exhauster truck significantly reduced the per empty cost. This indicates that clustering of empties and scaling up services is an important strategy for the sector going forward. Luckily, our stated preference study found evidence that households were amenable to emptying pits before they were full, and our revealed preference study found evidence that households positively respond to a discount for coordinated, simultaneous empties with one or more neighbors. This sort of coordinated scheduling of empties could be automated through ICT services: for example, an SMS-enabled marketing platform to pool demand through special offers to neighborhoods and households around those who are ready to empty.

The cost models also revealed that capital expenditure on trucks and equipment is a small portion of the total cost of service, when amortized and averaged over all empties. Therefore, providing such ‘bulky’ items as a type of subsidy will likely do little to reduce the prices. This is unfortunate, since the piped water and sewered sanitation services in large cities in low income countries are often subsidized in this way. Innovative subsidy mechanisms may be required if a similar level of support is to be given to FSM, especially when it comes to FS collection services.

Our revealed preference models show that households place a premium on improved pit emptying services. We estimated that, at the current level of market penetration for emptying services, the WTP for Pit Vidura’s improved services was US$79. This exceeded the average cost of emptying by US$27—a mark-up of 52%. If the goal is to convert all of the current empties to improved empties, and then this might be possible with an average price of US$79. If Pit Vidura could maintain an average of three to four empties per day while using the exhauster truck, it should be able to cover its costs. If instead the goal is to decrease the rate of sealing, as well as replace informal emptying, then some level of subsidy might be required.

Our study highlights the sizable gap that exists between the household willingness to pay and the cost of safe services. According to our estimates, only 15% of households currently choose to empty when their pits are full. Pit Vidura is well poised to improve services for those households, and it should be able to do so on its own if it averages between three and four empties per day. However, for the other 85% of households, this price might remain out of reach, or it might not be incentive enough for them to choose emptying over sealing. This might change in the future as space constraints slowly force them to choose emptying over sealing, although the magnitude of the impact on demand remains unclear. According to our revealed preference modeling, in order make emptying affordable for all households, at the present time prices may need to be reduced to roughly US$24. At this price, Pit Vidura would not be self-sufficient.

If Pit Vidura were able to regularly empty four pits per day using the exhauster truck, the average cost per pit would be reduced to US$61 per pit, according to our cost estimates. A price of US$24 would cover roughly 40% of this cost—a significant portion. Assuming Pit Vidura operates at optimal operational efficiency, bridging the remaining gap in WTP could be achieved through some kind of subsidy, either a cross subsidy or a direct subsidy to the consumer. Kigali might preserve environmental health, protect sanitation workers, and reduce sanitation inequities for all low-income residents, while making substantial progress towards SDG 6.2, if subsidies are provided for 60% of the cost of services, and Pit Vidura is able to empty four pits per day with the exhauster truck.

Subsidies need not come from general government receipts—cross subsidies might also help to smooth costs across time and over all members of the community. In our market assessment, we estimated that the average frequency of filling for pits was 8.7 years. Based on our cost modeling for four empties per day while using an exhauster truck, and our revealed preference modeling of demand, an average subsidy of US$37 per empty would be needed in order to make improved emptying accessible to all households. Charging all households US$0.36 per month would cover the cost of the subsidy and incentivize households to use the benefit that they had previously paid into. Setting up revenue collection services for of a monthly sanitation charge is logistically difficult and expensive, but such infrastructure is already in place for solid waste management in Kigali. Households report that they regularly pay the city US$2 per month for solid waste collection, even in the areas in which we conducted this study. Our study indicates that it might be worth exploring the possibility of adding a FS surcharge of US$0.36 to that amount, provided that those funds could be properly ring-fenced and entirely directed back towards FS collection subsidies.

Our stated preference results do not correspond with our estimates for current market prices for sealing and emptying. Our WTP for sealing is US$152 per pit, but this is the premium for the attribute of sealing as compared to informal, manual emptying, meaning that our model indicates the total WTP for sealing (including digging a new pit) is at least double the average rate found in our market assessment. While we took pains to limit the reporting bias as much as possible through our study design, collection instrument design, and during our data collection process, it is the most likely explanation for this discrepancy. Having said that, it is unlikely that reporting bias would influence the preference for different attributes differentially; the relative values of each attribute should still be valid. Our model estimates indicate that preferences for treatment slightly exceed the preferences for sealing, while those for worker protections and distant disposal are half as strong as those for treatment. Having said that, strong preferences and a non-zero WTP for services that protect workers, treat FS, and dispose of the treated FS at a distance from the home, are evident.

## 5. Conclusions

Our WTP study shows that even low-income households value protections for workers and the environment; even when pocketbooks are pinched, there is a non-zero WTP for social goods and environmental health. Our stated preference and revealed preference models back this up, and show that private individuals, through their choices, can, and perhaps should, financially participate or otherwise, in the design and provision of urban environmental services. Private participation on its own will not provide full coverage of improved FSM to all households in Kigali that use onsite systems; but, according to our estimates it might get us almost half-way there. That is significant and suggest that public bodies in Kigali should pursue a mechanism to meet households half-way, in its efforts to protect environmental health and expand access to safely managed sanitation.

## Figures and Tables

**Table 1:** Observed prices for Fecal Sludge (FS) collection services in informal settlements of low-income countries.

Source	Location	Emptying	Price per Empty
van Dijk M.P. et al. 2014 [6]	Dar Es Salaam, Tanzania	Manual	US$31.76–US$44.46
		Mechanized	US$63.52–US$76.22
Murungi C, van Dijk MP 2014 [7]	Kampala, Uganda	Manual	US$11.88–US$39.61
		Mechanized	US$19.81–US$59.42
Isunju JB, et al. 2013 [8]	Kampala, Uganda	Manual	US$8.5–US$17.2
		Mechanized	US$25.7–US$42.9
Frenoux & Tsitsikalis, 2015 [9]	Phnom Penh, Cambodia	Manual	US$25–US$30
		Mechanized	US$30–US$50

**Table 2:** Estimates from the research literature of willingness to pay (WTP) for FS collection in low-income countries.

Source	Location	Unit	Price per Unit
Balasubramanya et al., 2017 [10]	Bhaluka, Bangladesh	Entire Pit	US$6
Frenoux & Tsitsikalis, 2015 [9]	Phnom Penh, Cambodia	m^3^	US$7
Jenkins, Cumming, & Cairncross, 2015 [11]	Dar Es Salaam, Tanzania	300 liters	US$26
Harder, Sajise, & Galing, 2013 [12]	Dagupan, Phillipines	per month	US$0.90

**Table 3:** Prices were expressed on the coupons as per liter and per 2000 liters (as an example total price per empty). Prices presented here per 1200 liters (1200 liters was the average empty volume during the study).

Price Version	Single Empty Price (‘000 RwF)	Group Discount (‘000 RwF)	Group Empty Price (‘000 RwF)	Single Empty Price (US$)	Group Discount (US$)	Group Empty Price (US$)
1	24	6	18	26.67	6.67	20.00
2	24	12	12	26.67	13.33	13.33
3	36	6	30	40.00	6.67	33.33
4	36	12	24	40.00	13.33	26.67
5	48	6	42	53.33	6.67	46.67
6	48	12	36	53.33	13.33	40.00
7	60	6	54	66.67	6.67	60.00
8	60	12	48	66.67	13.33	53.33
9	72	6	66	80.00	6.67	73.33
10	72	12	60	80.00	13.33	66.67
11	84	6	78	93.33	6.67	86.67
12	84	12	72	93.33	13.33	80.00

**Table 4:** Attributes and discrete choice options used in the follow-up WTP surveys. Each attribute had two service levels that were associated with it, as listed below, with the exception of price, which had six levels.

Categorical Attributes and Levels		
	Discrete choice “0”	Discrete choice “1”
Pit fullness	Emptied before completely full, group discount applied	Emptied only when completely full, no group discount applied
Formality of services	Services provided by ‘builders’ (informal day laborers) using buckets and shovels	Services provided by a branded, registered business, with workers wearing uniforms and using protective gear
Speed of response	Empty performed within three weeks of request	Empty performed within one week of request
Distance of Disposal	Sludge is disposed inside of your cell ^1^, but outside of your household’s compound	Sludge is disposed outside of your cell2, in an open, public area in Kigali
Treatment	Sludge is not treated before disposal (treatment is a process that removes all smells and pathogens)	Sludge is treated before disposal (treatment is a process that removes all smells and pathogens)
Prices		
Volumetric Prices	15, 20, 30, 40, 50, 60 (RwF/Liter)	
Volumetric Group Discounts	5, 10 (RwF/Liter)	
Flat fees	20k, 40k, 45k, 60k, 80k, 90k,100k, 120k (RwF/empty)	

^1^ Cells are the smallest geographically denominated government administration unit. In Kigali they are roughly the size of an urban neighborhood.

**Table 5:** Cost categories for both the Flatbed Truck and Exhauster Truck scenarios.

Cost Type	Cost Categories	Flatbed Truck Costs	Exhauster Truck Costs
Opex	Maintenance and Consumables	Emptying equipment maintenance, replacing safety gear, cleaning/disinfecting equipment, storage depot	Emptying equipment maintenance, replacing safety gear, cleaning/disinfecting equipment, storage depot, truck maintenance
	Transportation	Flatbed truck rental, dumping fees	Fuel, dumping fees
	Labor	Collection crew	Collection crew, truck driver
Overhead	Capital	eVac pumps, trash removal tools, personal protective equipment	eVac pumps, trash removal tools, personal protective equipment, exhauster truck
	Rent	Office space, field depots	Office space, field depots
	Office Supplies	Stationary, internet	Stationary, internet
	Labor	Office Staff	Office Staff
	Advertising and Marketing	Printed marketing materials, radio advertisements, sales commissions, weekly text messages	Printed marketing materials, radio advertisements, sales commissions

**Table 6:** Market Analysis: summary statistics from survey data regarding pits, pit maintenance practices, and costs.

Survey Variable	Mean	95% CI	N
Household Size	6.1	±0.46	125
Residence time (years)	17.8	±2.89	110
Frequency of filling (years)	8.7	±0.85	266
Age of Latrine (years)	10.1	±1.87	94
Household responsible for pit maintenance	64%	±2.9%	1078
Pit was full before	43%	±3.1%	976
Pit has been emptied before	12%	±5.6%	131
Cost of sealing (all types) (US$)	109	±51.3	15
Cost of emptying (US$)	52	±14.0	35
Cost of solid waste collection (US$/Month)	2	±0.3	115

**Table 7:** Cost of delivering pit-emptying services using a rented, flatbed truck. The truck used during the trial has the capacity to hold 100 barrels (5000 liters), sufficient volume to handle up to two typical household empties.

Cost of Service—Rented, Flatbed Truck	1 Pit/Day	2 Pits/Day
Truck Rental (including fuel and driver)	US$54.00	US$27.00
Emptying staff	US$21.00	US$10.00
Sales Commission	US$1.80	US$1.80
Maintenance and Consumables	US$4.70	US$2.40
Dumping Fee	US$5.50	US$2.80
Total Opex per Empty	US$87.00	US$44.00
Amortized Capital Costs (Emptying equipment)	US$3.20	US$1.60
Management staff	US$52.00	US$26.00
Advertising	US$41.00	US$21.00
Rent and office supplies	US$7.30	US$3.60
Total Overhead per Empty	US$104.00	US$52.00
Total Costs per Empty	US$191.00	US$96.00

**Table 8:** Cost of pit-latrine emptying services delivered using an operator owned truck. The exhauster truck has the capacity of 10 m^3^, sufficient volume to handle up to four typical household empties.

Cost of Service—Owned, Exhauster Truck	2 Pits/Day	3 Pits/Day	4 Pits/Day
Fuel	US$17.00	US$11.00	US$8.30
Emptying staff (including driver)	US$16.00	US$21.00	US$14.00
Sales Commission	US$1.80	US$1.80	US$1.80
Maintenance and Consumables	US$12.00	US$8.00	US$6.00
Dumping Fee	US$3.00	US$2.00	US$1.50
Total Opex per Empty	US$50.00	US$44.00	US$32.00
Amortized Capital Costs (Truck and Equipment)	US$8.90	US$5.90	US$4.40
Management staff	US$26.00	US$17.00	US$13.00
Advertising	US$21.00	US$14.00	US$10.00
Rent and office supplies	US$3.60	US$2.40	US$1.80
Total Overhead per Empty	US$60.00	US$39.00	US$29.00
Total Costs per Empty	US$110.00	US$83.00	US$61.00

**Table 9:** Logistic regression results of revealed preference analysis. Dependent variable dichotomous (requested an empty = 1, no request = 0). Households were given coupons with randomly assigned volumetric prices. ‘Household responsible’ implies that households were fully or partially responsible for costs associated with pit maintenance (as opposed to the landlord having full responsibility).

HH Characteristic	Model 1	Model 2	Model 3	Model 4	Model 5
	Dependent variable:				
	Any Empty	Group Empty	Any Empty	Any Empty	Group Empty
Individual Price per empty (US$)	−0.036 *** (0.008)	−0.036 *** (0.011)	−0.035 *** (0.008)	−0.038 *** (0.009)	−0.041 *** (0.014)
Group Discount per empty (US$)		0.103 * (0.056)	0.084 ** (0.041)	0.070 (0.044)	0.067 (0.066)
Household Responsible			0.714 ** (0.328)		0.545 (0.525)
Pit Full and Almost Full				1.399 *** (0.303)	2.128 *** (0.519)
Constant	−1.321 *** (0.338)	−3.214 *** (0.837)	−2.853 *** (0.660)	−2.820 *** (0.679)	−4.368 *** (1.126)
Observations	1176	1176	1078	1156	1065
AIC	447.195	275.133	412.195	364.585	186.890

* *p* < 0.1; ** *p* < 0.05; *** *p* < 0.01.

**Table 10:** Demand forecasts are for some key price levels, using Model 1. Demand forecasts were made in order to show the confidence intervals on demand for any type of request, whether individual or group services were requested (see supplementary materials Table S4 for forecasts of group empty requests).

Price (US$)	Demand Forecast	(95% CI)
115.8	0.4%	(0.03–4%)
79.0	1.5%	(0.21–9%)
44.8	5.0%	(1.28–17%)
24.2	10.0%	(3.69–24%)

**Table 11:** Model 9 is a multinomial logit model. Prices included in the model were US$ per empty, as calculated by an average empty of 1200 L.

Name	Model 9
Price per empty	−0.013 ***
Branded, worker protections	84.3 ***
Distant Disposal	89.7 ***
Empty when full	5.62
Faster Response	16.2
Flat Fee Charged	−48.1 ***
Sealed	152 ***
Treated	136 ***

*** *p* < 0.01.

## Supplementary Materials

The following are available online at https://www.mdpi.com/1660-4601/16/23/4738/s1, Figure S1: Example coupon, in Kinyarwanda, with English translation. Table S1: Additional Variables Tested: Additional variables tested for the revealed choice model included pit fill frequency, whether the household had dealt with a full pit before, whether they had emptied before and whether they had sealed before. Logistic regression results of revealed preference analysis. Dependent variable dichotomous (requested empty from Pit Vidura = 1, no request, or request for individual empty = 0). Households were given coupons with randomly assigned volumetric prices, and randomly assigned discounts for group empties. None of these variables were significantly correlated with the choice to request pit emptying services; Table S2: Additional Model Variations: Additional model variations, using the same variables presented in Table 4, including models 1, 3 and 4, with dependent variable being the request for emptying services. Logistic regression results of revealed preference analysis. Dependent variable dichotomous (requested empty from Pit Vidura = 1, no request, or request for individual empty = 0). Households were given coupons with randomly assigned volumetric prices, and randomly assigned discounts for group empties. ‘Household responsible’ implies that households were fully or partially responsible for costs associated with pit maintenance (as opposed to the landlord having full responsibility); Table S3: Group Emptying Models: Additional model variations, using the same variables presented in Table 4, including models 2 and 5, with dependent variable being the request for group emptying services. Logistic regression results of revealed preference analysis. Dependent variable dichotomous (requested group empty from Pit Vidura = 1, no request, or request for individual empty = 0). Households were given coupons with randomly assigned volumetric prices, and randomly assigned discounts for group empties. ‘Household responsible’ implies that households were fully or partially responsible for costs associated with pit maintenance (as opposed to the landlord having full responsibility); Table S4: Demand Forecasts for Individual Empties and Group Empties: Demand forecasts are for key prices, using Model 1 and Model 9. Demand forecasts were made in order to show the confidence intervals on demand, for all service requests as well as the mean and confidence intervals for forecasts of demand for group empty.
