# Supplementary material for: Costs and Willingness to Pay for Pit Latrine Emptying Services in Kigali, Rwanda
Source: Int J Environ Res Public Health. 2019 Nov 27;16(23):4738. doi: 10.3390/ijerph16234738 (PMC6926954; doi:10.3390/ijerph16234738)
Supplement: Supplementary file 1 [file ijerph-16-04738-s001.pdf]

Supplementary

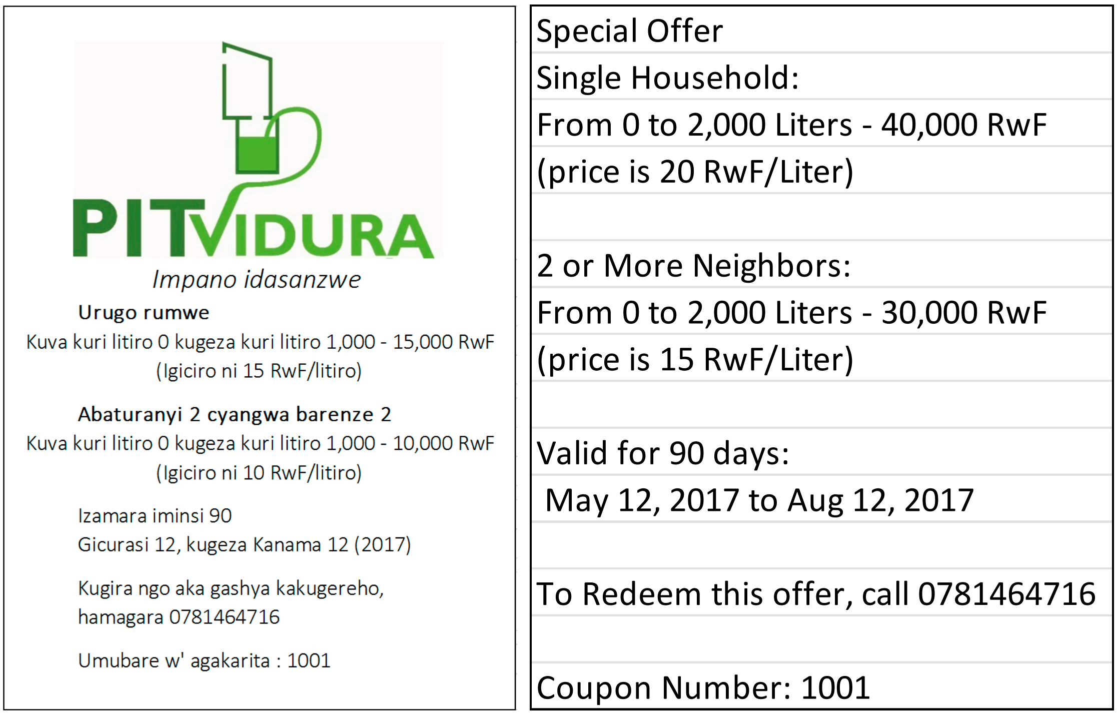

Figure S1. Example coupon, in Kinyarwanda, with English translation.

Table S1. Additional Variables Tested: Additional variables tested for the revealed choice model included pit fill frequency, whether the household had dealt with a full pit before, whether they had emptied before and whether they had sealed before. Logistic regression results of revealed preference analysis. Dependent variable dichotomous (requested empty from Pit Vidura = 1, no request, or request for individual empty = 0). Households were given coupons with randomly assigned volumetric prices, and randomly assigned discounts for group empties. None of these variables were significantly correlated with the choice to request pit emptying services.

	**Dependent variable**			
	Requested Emptying Services from Pit Vidura			
	Model A	Model B	Model C	Model D
Individual Price (US$)	-0.037** (0.016)	-0.039** (0.018)	-0.040** (0.018)	-0.052*** (0.013)
Pit fill frequency	0.033 (0.025)			
Emptied before		0.137 (0.583)		
Sealed before			0.372 (0.531)	
Pit filled before				0.406 (0.309)
Constant	-1.182** (0.570)	-1.644*** (0.602)	-1.836*** (0.675)	-1.585*** (0.415)
Observations	269	399	399	976
AIC	186.431	165.192	164.727	350.48

Note: * $p < 0.1$, ** $p < 0.05$, *** $p < 0.01$.

Table S2. Additional Model Variations: Additional model variations, using the same variables presented in Table 4, including models 1, 3 and 4, with dependent variable being the request for emptying services. Logistic regression results of revealed preference analysis. Dependent variable dichotomous (requested empty from Pit Vidura = 1, no request, or request for individual empty = 0).

Households were given coupons with randomly assigned volumetric prices, and randomly assigned discounts for group empties. 'Household responsible' implies that households were fully or partially responsible for costs associated with pit maintenance (as opposed to the landlord having full responsibility).

	Dependent variable			
	Requested Emptying Services from Pit Vidura			
	Model 6	Model 7	Model 8	Model 9
Individual Price (US$)	-0.038*** (0.009)	-0.035*** (0.008)	-0.039*** (0.009)	-0.038*** (0.009)
Group Discount (US$)	0.070 (0.044)			
Household Responsible		0.702** (0.327)		0.372 (0.340)
Pit Full & Almost Full	1.399*** (0.303)		1.426*** (0.302)	1.407*** (0.309)
Constant	-2.820*** (0.679)	-1.845*** (0.417)	-1.989*** (0.414)	-2.252*** (0.473)
Observations	1,156	1,078	1,156	1,065
AIC	364.585	414.503	365.184	354.184

Note: * $p < 0.1$, ** $p < 0.05$, *** $p < 0.01$.

Table S3. Group Emptying Models: Additional model variations, using the same variables presented in Table 4, including models 2 and 5, with dependent variable being the request for group emptying services. Logistic regression results of revealed preference analysis. Dependent variable dichotomous (requested group empty from Pit Vidura = 1, no request, or request for individual empty = 0). Households were given coupons with randomly assigned volumetric prices, and randomly assigned discounts for group empties. 'Household responsible' implies that households were fully or partially responsible for costs associated with pit maintenance (as opposed to the landlord having full responsibility).

	Dependent variable:			
	Requested Group Emptying Services from Pit Vidura			
	Model 10	Model 11	Model 12	Model 13
Individual Price (US$)	-0.036*** (0.011)	-0.035*** (0.011)	-0.043*** (0.014)	-0.041*** (0.014)
Group Discount (US$)	0.103* (0.056)	0.109* (0.059)	0.064 (0.066)	0.067 (0.066)
Household Responsible		1.061** (0.501)		0.545 (0.525)
Pit Full & Almost Full			2.218*** (0.517)	2.128*** (0.519)
Constant	−3.214*** (0.837)	−4.130 *** (0.972)	−3.984 *** (1.057)	−4.368 *** (1.126)
Observations	1176	1078	1156	1065
AIC	275.133	247.521	187.626	186.89

Table S4. Demand Forecasts for Individual Empties and Group Empties: Demand forecasts are for key prices, using Model 1 and Model 9. Demand forecasts were made in order to show the confidence intervals on demand, for all service requests as well as the mean and confidence intervals for forecasts of demand for group empty.

	**Model 1 (All Empties)**		**Model 10 (Group Empties)**			
Price (US$)	Demand Forecast	(95% CI)	Demand Forecast *(US$ 7.2 Discount)*	(95% CI)	Demand Forecast *(US$ 14.4 Discount)*	(95% CI)
115.8	0.40%	(0.03%–4%)	0.13%	(0%–13%)	0.27%	(0%–42%)

79	1.50%	(0.21%-9%)	0.49%	(0.01%-21%)	1.02%	(0.01%-56%)
44.8	5.00%	(1.28%-17%)	1.66%	(0.05%-31%)	3.41%	(0.05%-69%)
24.2	10.00%	(3.69%-24%)	3.41%	(0.17%-39%)	6.89%	(0.16%-76%)
